# Supplementary figures and images for: Phosphoregulation of DNA repair via the Rad51 auxiliary factor Swi5–Sfr1
Source: J Biol Chem. 2023 Jun 16;299(8):104929. doi: 10.1016/j.jbc.2023.104929 (PMC10366545; doi:10.1016/j.jbc.2023.104929)

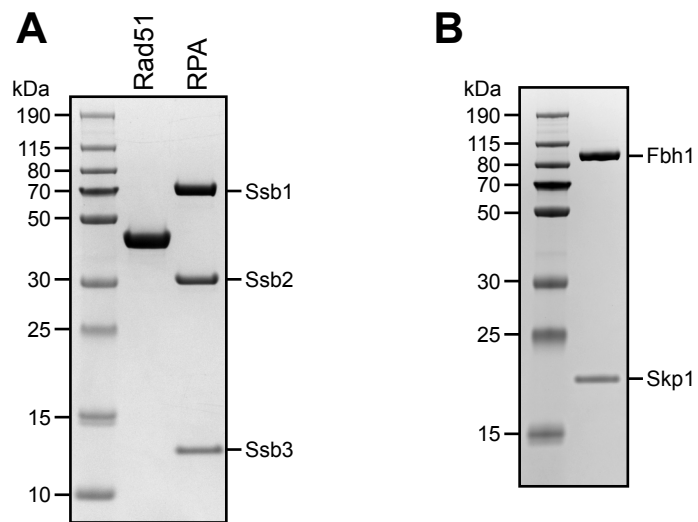

Supplement: Supporting Figure S1 — Purified proteins employed in this study.A and B, purified Rad51 and RPA (2 μg each) (A) or Fbh1–Skp1 complex (1 μg) (B) were analyzed by SDS-PAGE and Coomassie staining. RPA, replication protein A. [file mmc1.pdf]

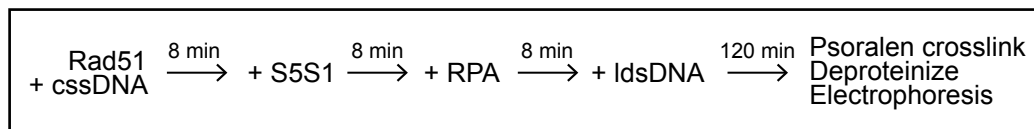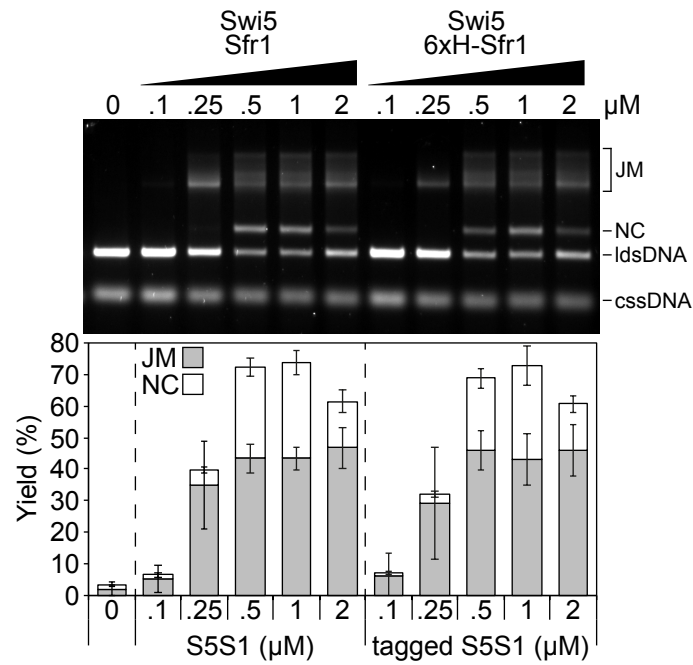

Supplement: Supporting Figure S2 — Hexahistidine-tagged S5S1 is proficient for the stimulation of Rad51-driven DNA strand exchange. Strand exchange assays were conducted as described for Figure 5B. Averages are plotted. n = 3, error bars represent standard deviation. [file mmc2.pdf]

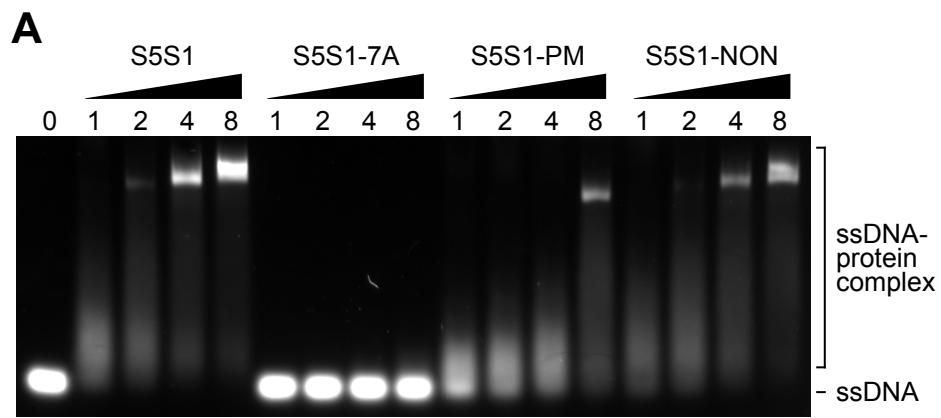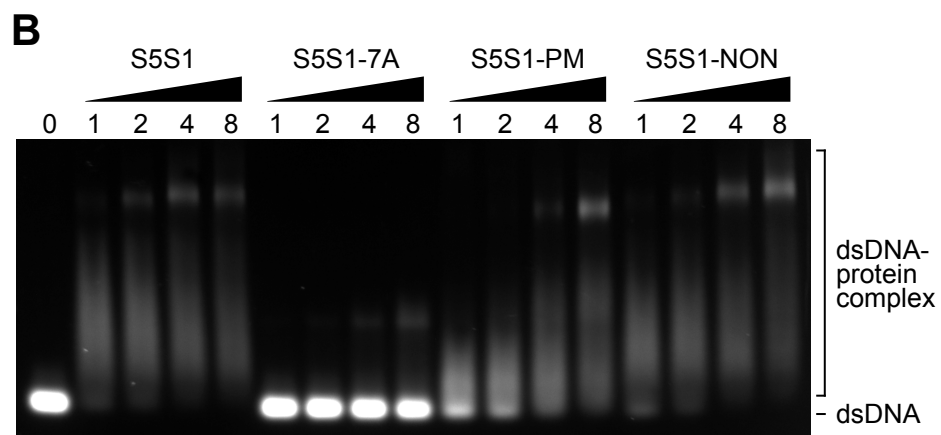

Supplement: Supporting Figure S3 — S5S1-PM is mildly impaired for DNA binding.A and B, electrophoretic mobility shift assays were conducted with 80-mer ssDNA (A) or dsDNA (B). DNA substrates (20 nM) were incubated with the indicated concentration of a S5S1 variant at 37 °C, and protein–DNA complexes were resolved by agarose gel electrophoresis. dsDNA, double-stranded DNA; ssDNA, single-stranded DNA. [file mmc3.pdf]
