# Supplementary material for: Phosphoregulation of DNA repair via the Rad51 auxiliary factor Swi5–Sfr1
Source: J Biol Chem. 2023 Jun 16;299(8):104929. doi: 10.1016/j.jbc.2023.104929 (PMC10366545; doi:10.1016/j.jbc.2023.104929)
Supplement: Supporting Figure S4 — Evolutionary conservation of phosphosites.A–C, alignment of S. pombe Sfr1 with Sfr1 orthologs from the Schizosaccharomyces genus (A), mouse (B), or humans (C). The five S. pombe phosphosites are shown in bold and Sfr1N is underlined. Alignments were performed in Clustal Omega. [file mmc4.pdf]

**A**

```
     S.pombe MSQ-TINSELNENATSQCKEDLKVSLSESDLRDSQ-------GQL---------GIENPP 43
S.japonicus ------------------------MSETNINDTTAKPQEEISSSTDILANVEEKSSIPV 35
S.octosporus MEKGEDNTELGI--------EADVNLTGSALIDNNSKQSEEAQNI---------NTELPP 43
S.cryophilus MENSEKNLELES--------EADSYLSRPAIDDINSIHSEEAQIS---------KTELPP 43
                                        ::   : *                          . *

     S.pombe KCNNS--GNHSDNLGFIEQSETVHPENEKALTPDLRDT-KIHTSLPITTPFSKKRAREAK 100
S.japonicus EPLEPVHVEDENNVPVPPK------DTPVMNTPEPKALHKLPDQQLFTPPISRKRLRDVK 89
S.octosporus TPSRD-------DPELKP--TTPNISQDNSDLPSLSDMEKHNPNAPLNPPNPRKRAREAK 94
S.cryophilus TPSRD-------NSELKP--TTPNASQDSSDLPSLSNIEKHTTNTQLNPPNPRKRAREAK 94
               .        :  .         .      *.     *   .  :. *  :** *:.*

     S.pombe NILLKPFKSPLRQ-TASPQVADTNLK----PSLAVTNLNSDETNTSSEPVTSPLRTTPNS 155
S.japonicus STLFKPFKSPVMKLPLTPQNQNTQSPCAHETPISASEELSSSSDLL---SSSPSKASFVR 146
S.octosporus SVLLKPFKSPLKY-PATPKSTPGTSK----VSNYTSDELKSSDEVSSDPVSSPSRPPPRV 149
S.cryophilus SVLLKPFKSPLKY-PATPKTTPGTCK----VANYASDELKSSDDVLSDPISSPSRPSPRV 149
             . *:******:     :*:                 .::  ... :       :** :

     S.pombe IKRQKRLFKSPISNCLNPKSDPEITQLLSRRLKLEKEVRNLQEQLITAETARKVEAKNED 215
S.japonicus KKRVKRSFQSPIAS-LKSSSDPELVALLSHRLRVEKEVKTLQDDLNTAEAAFKVETKDED 205
S.octosporus IKRAKKTFRSPVSSGSKVQSDPEVARLLSERLILEKDVRNLQEQLTTAETAKKVESKNED 209
S.cryophilus IKRAKKVFRSPISATNKVQNDPEVARLLSERLKLEKDVRNLQEQLTTAEAAVKVELKNED 209
              ** *: *:**::   : ..***:. ***.** :**:*:.**::* ***:* *** *:**

     S.pombe KDLQTLIQKWKNAAQQAAEVLFKPMAERIRLAGGVTQSFRIEEGENKGQIQEVRTEFTMS 275
S.japonicus DDLIVLIHRWRAAAQQAADVLFHPMAERIANAGGVTRCVRLQQDG-QEYVSEEKTKYTMG 264
S.octosporus KDLEALVYKWRGVAQRAAQVLYQPMAERIRLAGGVMQKYTIQEGEDAGQIEEKRTEFTMG 269
S.cryophilus KDLEGLVFKWRGVAQRAAQVLYRPMAERIRLAGGVTQTYTIQEGENKGNIEETRSEFSMG 269
             .**  *: :*: .**:**:**::******  **** :   :::.     :.* :::::*.

     S.pombe MFLNQFGVPVHLMSFDEENGDWKS-  299
S.japonicus MFLQQFGVPYELIGYDPEEDDWTTT  289
S.octosporus MFLHQFGVPFDLIAYDEELEDWKN-  293
S.cryophilus MFLQQFGVPFDLIAYDEEFEDWKG-  293
             ***:***** .*:.:* *  **.
```

**B**

```
     S.pombe MSQTINSELNENATSQCKEDLKVSLSESDLRDSQGQLGIENPPKCNNS---GNHSDNLGF 57
 M.musculus MAEEGNQEF----TSK----MENS-SDSASTSPDAPQPSENPPSPPTSPAAPQTSENPPS 51
             *::  *.*:    **:    :: * *:*   . :.    ****.  .*    : *:*

     S.pombe IEQSETVHPENEKALTPDLRDTKIHTSLPITTPFSKKRAREA-----KNILLKPFKSPLR 112
 M.musculus --------P----PTSPAVPQTRENPPSPPTSPAAPQPRENPPSPPTSPAAPQPRENPPS 99
                     *      :* : :*: :   * *:* : :  .:      .    :* :.*

     S.pombe ---QTASPQVAD---------TNLKP--------------------SLAVTNLNSDET- 138
 M.musculus PPTSPAAPQPRENPPSPHSNSSGKQPLSGTPKERLKKARSSSHSFCSVVKRMKVENDENN 159
                . *:** :          :. :*                   :   :::.**.

     S.pombe NTSSEPVTSPLRT----TPNSIKRQK----RLFKSPISNCLNPKSDPEIT---------Q 181
 M.musculus ETLSEPGESSKEENCSKAQESLKNKDSEPGEKSSEEKNTCESKSSDTGSSNALPKESENA 219
             :* ***  *  .     : :*:*.:.    .  ..  ..* . .**    :

     S.pombe LL-----SRRLKLEKEVRNLQEQLITAETARKVEAKNEDKDLQTLIQKWKNAAQQAAEVL 236
 M.musculus IIREKLKQEKIRLIRQVEEKEDLLRRLKLVKMYRIKNDVTELENLIKKWRKCGQRLLCEL 279
             ::     ..:::* ::*.: :: *   : .:  . **: .:*:.**:**::..*:    *

     S.pombe FKPMAERIRLAGGVTQSFRIEEGENKGQIQEVRTEFTMSMFLNQFGVPVHLMSFDEENGD 296
 M.musculus QSIMSED------------------------EDEKLTLTELIDFYGIDDNLLHYNRSEEE 315
              . *:*                         ::*:: ::: :*:  :*: ::..: :

     S.pombe WKS-  299
 M.musculus FTGV  319
             :..
```

**C**

```
     S.pombe MSQTINSELNENATSQCKEDLKVSLSESDLRDSQGQLGIENPPKCNNSGNHSDNLGFIEQ 60
  H.sapiens ---------------------------------------------MAEGEKNQDFTFKME 15
                                                         .*::.::: *  :

     S.pombe SETVHPENEKALTPDLRDTKIHTSLPITTPFSKKRAREAKNILLKPFKSPLRQTASPQVA 120
  H.sapiens S----PSDSAVVLPSTPQASANPS----SPYTNSSRKQP---MSATLRERLRKTRFSFNS 64
             *    *.:. .: *.  ::. : *    :*:::.  ::    :   ::. **:*     :

     S.pombe DTNLKPSLAVTNLNSDETNTSSEPVTSPLRT---TPNSIKRQKRLF------K---SPIS 168
  H.sapiens SYNVVKRLKVESEENDQT-FSEKPASSTEENCLEFQESFKHIDSEFEENTNLKNTLKNLN 123
             . *:   * * . :.*:*  *.:*.:*.:*  ..     :*:*: .  *       *   . :.

     S.pombe NCLNPKSDPE----------ITQLL-----SRRLKLEKEVRNLQEQLITAETARKVEAKN 213
  H.sapiens VCESQSLDSGSCSALQNEFVSEKLPKQRLNAEKAKLVKQVQEKEDLLRRLKLVKMYRSKN 183
              * . . *           :*       :.: ** *:*:: :: *   :  :.:  .:**

     S.pombe EDKDLQTLIQKWKNAAQQAAEVLFKPMAERIRLAGGVTQSFRIEEGENKGQIQEVRTEFT 273
  H.sapiens DLSQLQLLIKKWRSCSQLLLYELQSA-------------------------VSEENKKLS 218
             : .:** **:**:..:*      * .                          :.* ..:::

     S.pombe MSMFLNQFGVPVHLMSFDEENGDWKS-  299
  H.sapiens LTQLIDHYGLDDKLLHYNRSEEEFIDV  245
             :: :::::*:  :*: ::..: :: .
```
